# MicroRNA-126 Modulates Palmitate-Induced Migration in HUVECs by Downregulating Myosin Light Chain Kinase via the ERK/MAPK Pathway

**DOI:** 10.3389/fbioe.2020.00913

**Published:** 2020-07-31

**Authors:** Yi Wang, Mei Wang, Pei Yu, Li Zuo, Qing Zhou, Xiaomei Zhou, Huaqing Zhu

**Affiliations:** ^1^Department of Biological Engineering, School of Life Sciences, Anhui Medical University, Hefei, China; ^2^Laboratory of Molecular Biology and Department of Biochemistry, Anhui Medical University, Hefei, China; ^3^General Department of Hyperbaric Oxygen, Hefei Hospital Affiliated to Anhui Medical University, Hefei, China

**Keywords:** microRNA-126, cell migration, myosin light chain kinase, endothelial dysfunction, ERK/MAPK pathway

## Abstract

MicroRNA-126 (miR-126) is an endothelial-specific microRNA that has shown beneficial effects on endothelial dysfunction. However, the underlying molecular mechanism is unclear. The present study evaluated the effects of miR-126 on the cell migration and underlying mechanism in HUVECs treated with palmitate. The present results demonstrated that overexpression of miR-126 was found to decrease cell migration in palmitate-treated HUVECs, with decreased MLCK expression and subsequent decreased phosphorylated MLC level. miR-126 also decreased the phosphorylation of MYPT1 in palmitate-treated HUVECs. In addition, it was demonstrated that miR-126 decreases expression of the NADPH oxidase subunits, p67 and Rac family small GTPase 1 with a subsequent decrease in cell apoptosis. Moreover, the phosphorylation of ERK was reduced by miR-126 in palmitate-induced HUVECs. Taken together, the present study showed that the effect of miR-126 on cell migration and cell apoptosis is mediated through downregulation of MLCK via the ERK/MAPK pathway.

## Introduction

Atherosclerosis (AS) is a chronic progressive pathological process characterized by multiple factors. Specifically, endothelial dysfunction is the earliest step in the pathogenesis of AS (Gimbrone and García-Cardeña, 2016). Palmitate, a main component of saturated fat, is associated with increased cardiovascular disease risk. Furthermore, clinical and experimental studies have demonstrated that high concentrations of free fatty acid (FFA) in the plasma, promotes endothelial dysfunction (Arijit et al., 2017). Palmitate increased monocyte expression of CD11b, which was associated with increased adhesion to rat aortic endothelium and CD36 expression, which promoted oxidized LDL uptake (Gao et al., 2012).

The phosphorylation of myosin regulatory light chain (MLC) has an essential role in the control of actomyosin contractility participates in cell contraction, cell adhesion, cell migration and epithelial barrier formation. Myosin light chain kinase (MLCK) induced the phosphorylation of MLC which activated by Ca^2+^-calmodulin, is important in stress fiber formation and cell contractility. Aberrant expression of MLCK was shown to promote the progression of numerous inflammatory diseases, including pancreatitis, respiratory diseases, cardiovascular diseases, cancer and inflammatory bowel disease (Kim et al., 2012; Yu et al., 2018; Wang et al., 2019).

MicroRNAs (miRNAs) are a class of small 18–22 nucleotide, non-coding, single-stranded RNA molecules that regulate gene expression at the post-transcriptional level by binding to target mRNA. It was well-known that abnormal expression of miRNAs have been closely linked to the progression of AS by regulating endothelial cell function, lipid accumulation and vascular cells proliferation (Wojciechowska et al., 2017; Hung et al., 2018). Furthermore, miR-126, a miRNA specific for endothelial cells mediates vascular development and angiogenesis. Circulating level of miR-126 was decreased in the coronary artery disease (CAD) patients compared with healthy control (Wang et al., 2017). Previous study has shown that miR-126 play a protective role in human cardiac microvascular endothelial cells from hypoxia/reoxygenation-induced injury and inflammatory response by increasing NO secretion (Yang et al., 2017). In addition to vascular changes, miR-126 also modulates inflammation, and regulate lipid metabolism in endothelial cells (Yuan et al., 2016). Our previous study showed that miR-126 serves an anti-apoptotic role in palmitate-treated human umbilical vein endothelial cells (HUVECs) by decreasing the production of reactive oxygen species (ROS) (Wang et al., 2015). However, the role of miR-126 in the MLCK expression is unclear. Therefore, this study was aimed to analyze the effect exerted by miR-126 in MLCK expression in palmitate treated HUVECs, as well as the underlying mechanisms.

## Materials and Methods

### Reagents and Antibodies

Palmitate and oleate were obtained from Sigma-Aldrich. DMEM medium was obtained from Gibco. FBS was purchased from the Zhejiang Tianhang Biological Technology. Anti-MLCK, anti-MLC, anti-MYPT1, anti-p-MYPT1, anti-ERK, anti-p-ERK and anti-β-actin antibodies were purchased from Santa Cruz Biotechnology. Anti-pMLC was obtained from Cell Signaling Technology. Anti-NOXA2/p67phox and anti-Rac family small GTPase 1 were purchased from Abcam.

### Cell Culture and Transfection

HUVECs were cultured at 37°C in DMEM supplemented with 10% FBS at 5% CO_2_ incubator. miR-126 mimic, miR-126antagomir and a scrambled oligonucleotide (Qiagen GmbH) were transfected with TransMessenger Transfection Reagent (Qiagen GmbH) according to the instructions. After 24 h, cell medium was changed with palmitate or oleate supplementation for 24 h. Oleate, as an unsaturated fatty acid exerts beneficial effects on endothelial dysfunction, was used as a control (Grenon et al., 2012). Each experiment was repeated a minimum of three times.

### miR-126 Expression Assay

Total RNA from HUVECs was extracted by using TRIzol reagent (Invitrogen; Thermo Fisher Scientific, Inc.). A miRNA plate assay kit (Signosis, Inc.) and an oligo mix specific for miR-126 (Signosis, Inc.) were used to detect miR-126 expression following the manufacturer’s protocol. The U6 small nucleolar RNA was chosen as an endogenous reference of miR-126. miR-126 expression was also assessed by reverse transcription-quantitative polymerase chain reaction (RT-qPCR), the sequences of the primers were as follows: miR-126, forward 5′-UCGUACCGUGAGUAAUAAUGCG-3′, reverse 5′-CAUUAUUACUCACGGUACGAUU-3′, and U6, forward 5′-CTCGCTTCGGCAGCACA-3′, and reverse 5′-AACGCTTCACGAATTTGCGT-3′. The reaction procedure was as follows: pre-denaturation at 95°C for 10 min, followed by 40 cycles of 95°C for 15 s, 65°C for 15 s, and 72°C for 15 s.

### Scratch Healing Assay

HUVECs were seeded in 12-well plates and transfected as indicated, a total of 24 h later, palmitate or oleate were incubated with HUVECs. When the cells reached confluence, a sterile 200-μl pipette tip was used to create a horizontal wound in the confluent monolayer. Photographs of scratch wounds were captured prior to stimulation (0 h) and 24 h after incubation. The initial distance (0 h) and the distance traveled by cells after 24 h as detected using a microscope (Olympus). The percentage of wound healing was calculated using Image J (National Institutes of Health).

### Cell Apoptosis

Cells underwent transfection for 48 h, followed by digestion and centrifugation to remove the supernatant. The cells were then washed with PBS and centrifuged again. The apoptotic rate of HUVECs was detected using annexin V-allophycocyanin apoptosis detection kit (Beyotime) following the manufacturer’s instructions.

### Western Blot Analysis

Protein samples were extracted from cultured cells. Cells were lysed using RIPA buffer. The protein concentrations were determined using a BCA protein assay kit (Beyotime Institute of Biotechnology). The protein was separated by SDS-PAGE, then transferred to polyvinylidene fluoride membranes. The membrane was blocked with 5% non-fat dry milk solution at room temperature for 2 h, and followed by incubation with the following primary antibodies (anti-MLCK, anti-MLC, anti-pMLC, anti-MYPT1, anti-pMYPT1, anti-ERK, anti-p-ERK, anti-p67phox, anti-Rac1, and anti-β-actin, all were used at a dilution of 1:1,000.) overnight. The 2 day, the membranes incubation with secondary antibodies at room temperature for 2 h and visualized with a Super Signal West Pico kit (Thermo Fisher Scientific, Inc.). Data were quantified through densitometry using Quantity One software.

### Statistical Analysis

All data are reported in the form of mean ± standard deviation. The significant differences among groups were carried out with ANOVA followed by Newman-Keuls test for multiple comparisons. *P*-values below 0.05 were considered to indicate a statistically significant difference. Statistical analyses were carried out using SPSS 17.0.

## Results

### miR-126 Reduces Palmitate-Induced Migration (motility) in HUVECs

**FIGURE 1:**
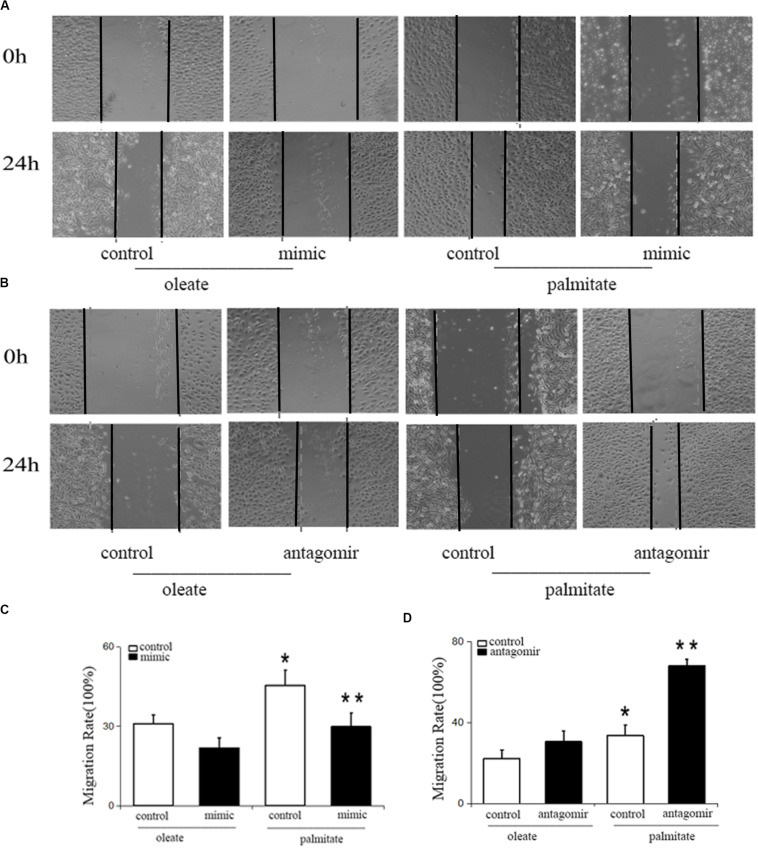
Effect of miR-126 on the migration of palmitate-treated HUVECs. **(A,C)** The migration rate was increased in palmitate-treated HUVECs compared with oleate-treated HUVECs, miR-126 mimic inhibited the migration rate of palmitate-treated HUVECs. **(B,D)** miR-126 antagomir increased the migration rate of palmitate-treated HUVECs. *P < 0.05 vs oleate-treated HUVECs; **P < 0.05 vs palmitate-treated HUVECs.

To study the influence of miR-126 on cell migration in palmitate-treated HUVECs, the present study performed wound healing scratch assays. Following treatment with palmitate, the migration distances of HUVECs were found to be significantly longer compared with the oleate-treated HUVECs 24 h after injury. However, miR-126 mimic decreased the migration distances in palmitate-treated HUVECs compared with the control group (transfected with a scrambled oligonucleotide). By contrast, miR-126 antagomir further increased the migration distances in palmitate-treated HUVECs compared with the control group (Figure 1). The results indicated that miR-126 reduced cell migration in palmitate-treated HUVECs.

### miR-126 Reduces MLC Phosphorylation by Regulating the Expression of MLCK and MYPT1 Phosphorylation in Palmitate-Treated HUVECs

**FIGURE 2:**
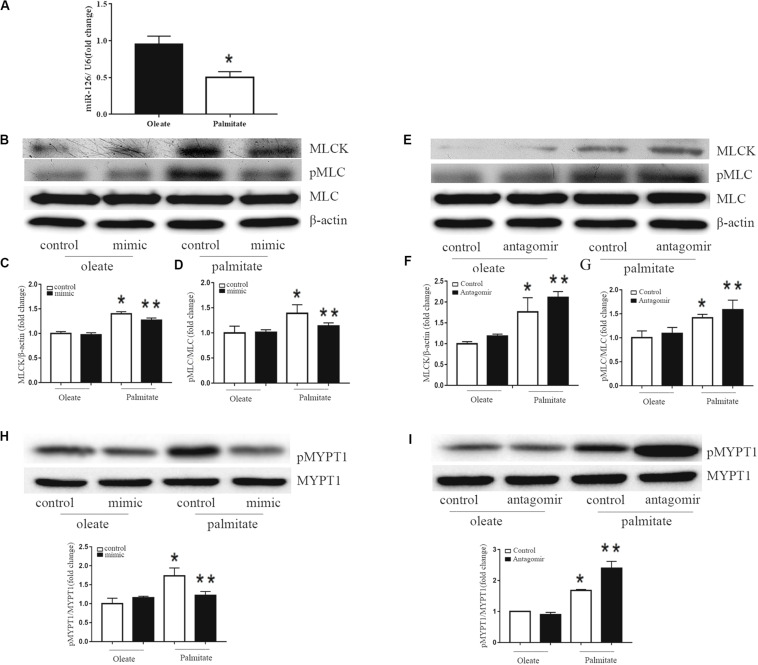
Effect of miR-126 on the expression of MLCK, p-MLC and p-MYPT1 in palmitate-treated HUVECs. **(A)** Palmitate inhibited the expression of miR-126 in HUVECs compared with oleate. **(B–D)** The expression level of MLCK was upregulated in palmitate-treated HUVECs compared with oleate-treated HUVECs, and the p-MLC/MLC ratio in palmitate-treated HUVECs was upregulated, which all inhibited by miR-126 mimic. **(E–G)** The expression of level of MLCK and p-MLC/MLC ratio in palmitate-treated HUVECs was enhanced by miR-126 antagomir. **(H)** The pMYPT1/MYPT1 ratio was upregulated in palmitate-treated HUVECs compared with oleate-treated HUVECs which inhibited by miR-126 mimic. **(I)** The pMYPT1/MYPT1 ratio in palmitate-treated HUVECs was enhanced by miR-126 antagomir. *n* = 3. *P < 0.05 vs oleate-treated HUVECs; **P < 0.05 vs palmitate-treated HUVECs.

To investigate the effect of palmitate on miR-126 expression in HUVECs, HUVECs were incubated with palmitate or oleate (0.1 mM) as a control. After 24 h, miR-126 expression was determined in HUVECs using the miRNA plate assay. As presented in Figure 2A that palmitate significantly decreased miR-126 expression. To measure the influence of miR-126 on MLCK expression, the level of MLCK was analyzed in palmitate-treated HUVECs transfected with a miR-126 mimic or miR-126 antagomir. Palmitate induced a markedly increased MLCK protein expression in HUVECs compared with oleate-treated cells. Furthermore, overexpression of miR-126 decreased MLCK protein expression in palmitate-treated HUVECs, while downregulation of miR-126 had the opposite effect (Figures 2B,C). In addition, MLCK is known to catalyze MLC phosphorylation. The present results demonstrated that the expression of MLC in palmitate-treated HUVECs was not significantly different compared with oleate-treated HUVECs, However, the phosphorylated MLC/MLC ratio in palmitate-treated HUVECs was increased compared with oleate-treated HUVECs, andmiR-126 mimic significantly decreased MLC phosphorylation (Figures 2B,D). By contrast, miR-126 antagomir significantly increased MLC phosphorylation (Figures 2E,G). To determine whether miR-126 regulates MLC phosphorylation through the phosphorylation of MYPT1, the levels of pMYPT1 and MYPT1 were quantified. The results demonstrated that miR-126 mimic significantly increased MYPT1 phosphorylation, whereas miR-126 antagomir significantly decreased MYPT1 phosphorylation (Figures 2H,I). These results indicated that miR-126 reduced the phosphorylation of MLC by regulate the expression of MLCK and the phosphorylation of MYPT1 in palmitate-treated HUVECs.

### miR-126 Reduces NADPH Oxidase Subunits Rac1 and P67phox Expression in Palmitate-Treated HUVECs

**FIGURE 3:**
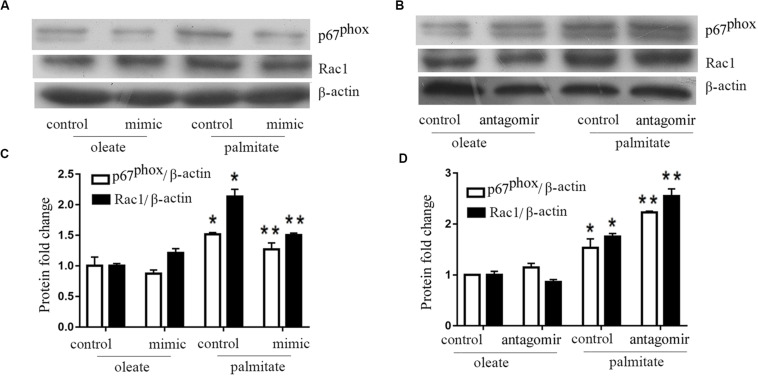
miR-126 modulates the expression of p67phox and Rac1 in palmitate-treated HUVECs. **(A,C)** The expression level of p67phox and Rac1 were upregulated in palmitate-treated HUVECs compared with oleate-treated HUVECs, miR-126 mimic inhibited the expression level of p67phox and Rac1 in palmitate-treated HUVECs. **(B,D)** miR-126 antagomir enhanced the expression level of p67phox and Rac1 in palmitate-treated HUVECs. *n* = 3. *P < 0.05 vs oleate-treated HUVECs; **P < 0.05 vs palmitate-treated HUVECs.

Previous studies have shown that NADPH oxidase activity was is influenced by MLCK. Therefore, NADPH oxidase expression levels were quantified in the present study. The results revealed that palmitate increased the expression levels of Rac1 and p67phox (subunits of NADPH oxidase 2) in HUVECs compared with oleate-treated HUVECs (Figure 3). Furthermore, overexpression of miR-126 decreased Rac1 and p67phox protein expression in palmitate-treated HUVECs, while downregulation of miR-126 had the opposite effect. These results indicated that miR-126 reduced the expression of Rac1 and p67phox in palmitate-treated HUVECs.

### miR-126 Reduces Apoptosis in Palmitate-Treated HUVECs

**FIGURE 4:**
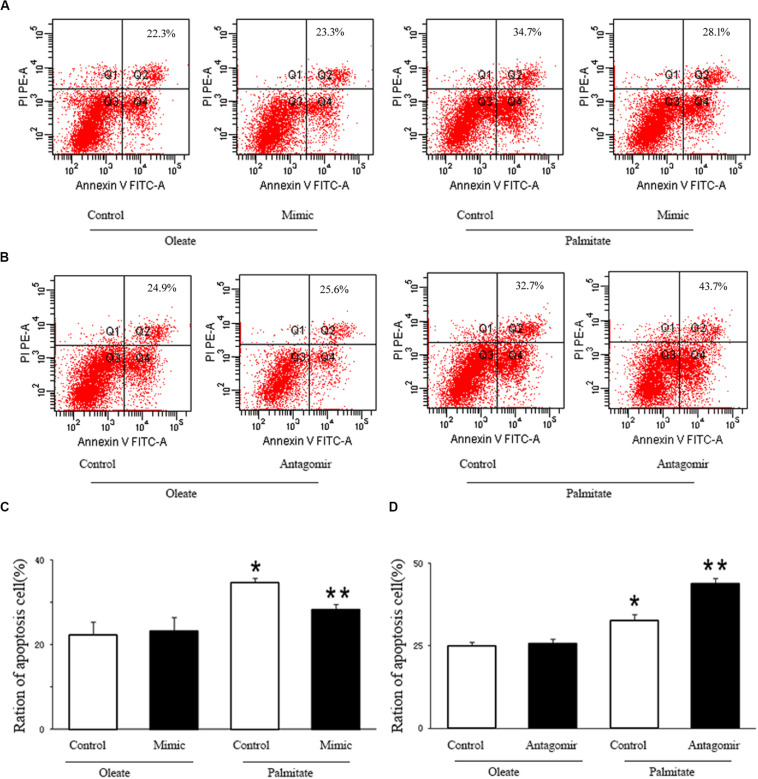
miR-126 affected palmitate-induced HUVECs apoptosis as assessed by FACS. **(A,C)** FACS was used to detect HUVECs apoptosis, HUVECs apoptosis rate was increased in palmitate-treated HUVECs compared with oleate-treated HUVECs, miR-126 mimic decreased cell apoptosis in palmitate-treated HUVECs. **(B,D)** FACS was used to detect HUVECs apoptosis, HUVECs apoptosis rate was increased in palmitate-treated HUVECs compared with oleate-treated HUVECs, miR-126 antagomir promoted cell apoptosis in palmitate-treated HUVECs. *n* = 3. *P < 0.05 vs oleate-treated HUVECs; **P < 0.05 vs palmitate-treated HUVECs.

We investigated the effect of miR-126 on the regulation of HUVECs apoptosis induced by palmitate. As presented in Figure 4, the number of apoptotic cells in palmitate-treated HUVECs was higher than those observed in oleate-treated HUVECs. However, upregulation of miR-126 significantly alleviated apoptosis in palmitate-treated HUVECs, while downregulation of miR-126 further increased the number of apoptotic cells in palmitate-treated HUVECs. The data indicated that miR-126 inhibited apoptosis in palmitate-treated HUVECs.

### miR-126 Attenuates Activation of ERK in Palmitate-Treated HUVECs

**FIGURE 5:**
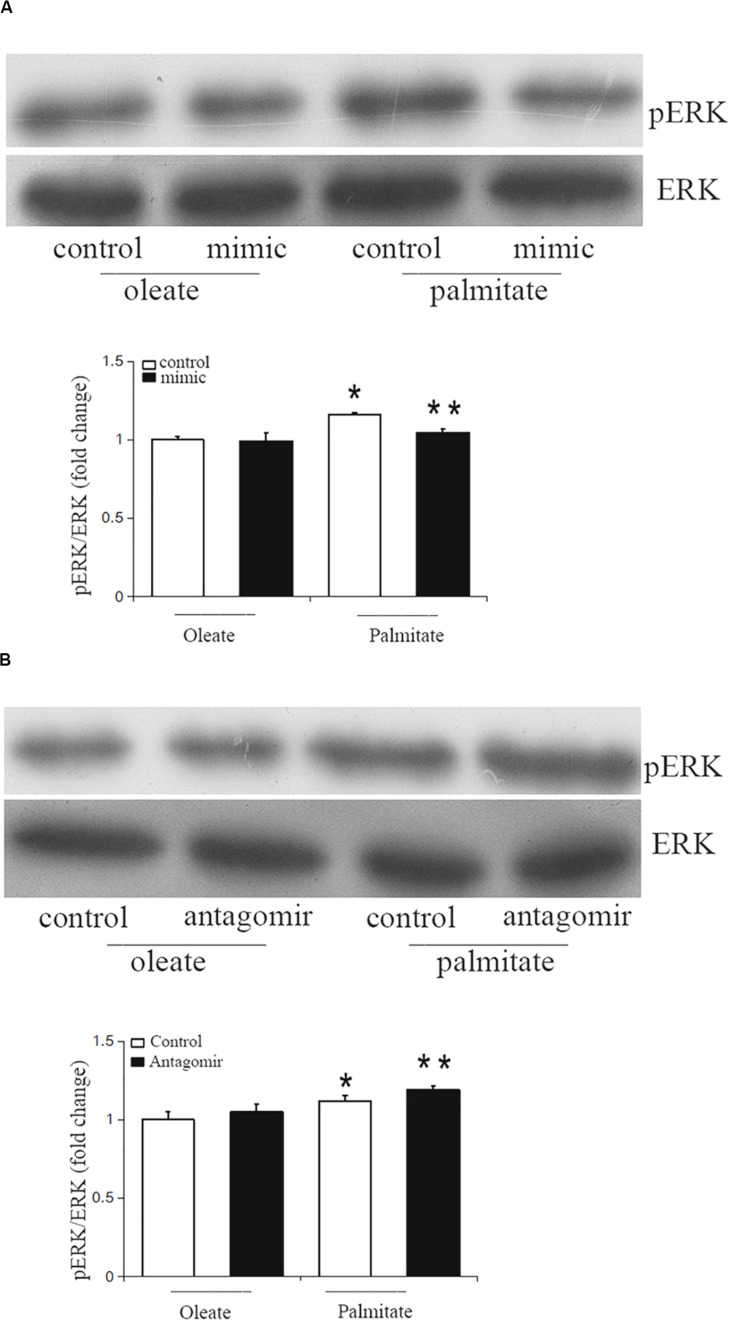
miR-126 modulates the phosphorylation of ERK in palmitate-treated HUVECs. **(A)** Western blot results showed that phosphorylated ERK/ERK ratio was upregulated in palmitate-treated HUVECs compared with oleate-treated HUVECs, miR-126 mimic decreased the phosphorylated ERK/ERK ratio in palmitate-treated HUVECs. **(B)** Western blot results showed that phosphorylated ERK/ERK ratio was upregulated in palmitate-treated HUVECs compared with oleate-treated HUVECs, miR-126 antagomir increased the phosphorylated ERK/ERK ratio in palmitate-treated HUVECs. *n* = 3. *P < 0.05 vs oleate-treated HUVECs; **P < 0.05 vs palmitate-treated HUVECs.

The ERK/MAPK pathway has been reported to be associated with endothelial dysfunction. Next, we investigated whether miR-126 participates in regulating the ERK/MAPK pathway in palmitate-induced HUVECs. miR-126 mimic, antagomir or a scrambled oligonucleotide were transfected into HUVECs, respectively, a total of 24 h later, HUVECs were exposed to palmitate or oleate (0.1 mM) for a further 24 h. As shown in Figure 5, phosphorylated ERK/ERK ratio was upregulated in palmitate-treated HUVECs compared with oleate-treated HUVECs, overexpression of miR-126 attenuated the phosphorylated ERK/ERK ratio in palmitate-treated HUVECs, whereas downregulation of miR-126 increased the phosphorylated ERK/ERK ratio in palmitate-treated HUVECs. The results indicated that miR-126 modulate activation of ERK in palmitate-treated HUVECs.

## Discussion

The endothelial cells (ECs) dysfunction in AS is characterized by increased cellular migration, apoptosis and enhanced permeability of the endothelial cell monolayer, allowing passage of lipids and inflammatory factors (Chistiakov et al., 2016; Hu et al., 2020). miR-126 is the most prominent miRNA in ECs, abnormal expression of miR-126 may contributing to the pathogenesis of AS. *In vivo* study showed that miR-126 is essential for maintaining vascular integrity by involved in endothelial cell migration, disruption of cytoskeletal structure and cell apoptosis (Huveneers et al., 2015). The present study, demonstrated that the presence of miR-126 ameliorated cell migration and cell apoptosis, and subsequently reduced the expression of MLCK in HUVECs that had previously been treated with palmitate.

Furthermore, the endothelial cytoskeleton contractile machinery has an important role in maintaining barrier properties of the endothelium (Kása et al., 2015). Additionally, MLCK is a specifically enzyme via catalyze the phosphorylation of MLC mediate the reorganization of the cytoskeleton, leading to the disruption of endothelial barrier integrity (Rossi et al., 2011). The compromised endothelial barrier become more permeable to lipids and immune cells, ultimately leading to AS lesion formation (Schnittler, 2016). The present results indicated that miR-126 attenuates the expression of MLCK and decreases phosphorylation of MLC in palmitate-treated HUVECs. Dephosphorylation of MLC is accomplished by MLCP, which is consisting of MYPT1, a myosin target subunit, and a subunit with uncertain function. Dephosphorylation of MLC results in cell relaxation. The catalytic activity of MLCP are inhibited by the phosphorylation of MYPT1 at multiple sites by several kind of kinases, therefore, led to decreased dephosphorylated MLC and thus, vascular smooth muscle contraction (Qiao et al., 2014; Chang et al., 2016; Gao et al., 2017; Deng et al., 2020). The current study found that miR-126 decreased phosphorylation levels of MYPT1 in palmitate-treated HUVECs, which may account for the observed decrease in MLC phosphorylation. Therefore, miR-126 may modulate the phosphorylation of MLC via the regulation of MLCK expression and MLCP activity.

Previous studies have found that knockout of MLCK reduces the level of oxidized low-density lipoprotein (oxLDL)-induced endothelial hyper-permeability and also reduced the size of aortic lesions. Notably, it was also identified that MLCK acts through both MLC phosphorylation-coupled and -uncoupled pathways (Shen et al., 2010). Furthermore, Usatyuk et al. demonstrated that upregulation of MLCK in human pulmonary artery endothelial cells enhances the activation of endothelial NADPH oxidase and enhances ROS production (Usatyuk et al., 2012). Alternatively, downregulation of MLCK significantly inhibits NADPH oxidase, resulting in subsequently reduced ROS production. NADPH oxidase are important enzymes that regulate ROS generation in the vasculature. NADPH oxidase inhibitors have been shown to attenuate palmitate-induced excessive production of ROS within animal models (Li et al., 2019). Therefore, it was postulated that miR-126 could regulate NADPH oxidase expression by the regulation of MLCK. The current study examined the level of cellular apoptosis, as well as the expression of NADPH oxidase 2 subunits, such as p67phox and Rac1. The results indicated that miR-126 ameliorated cell apoptosis and reduced expression of p67phox and Rac1 in palmitate-treated HUVECs. These results were consistent with our previous study showing that miR-126 reduced ROS production in palmitate-treated HUVECs (Wang et al., 2015).

The MAPK signaling pathway has a vital function in the pathogenesis of AS. Chandra S et al. reported that high glucose induced endothelial dysfunction are mediated by the ERK/MAPK pathway (Chandra et al., 2019). Recently, a study have demonstrated that the ERK/MAPK pathway contribute to the activation of MLCK (Tan et al., 2014). Therefore, a previous study has shown that overexpression of miR-126 suppressed ERK pathway activity in glioma cells and resulted in the inhibition of glioma cell proliferation and invasion (Li et al., 2017; Wang et al., 2020). In the current study, we found that miR-126 could affect the activity of MLCK contributed to endothelial dysfunction via the ERK/MAPK pathway. The present data revealed that ERK activation was decreased in the presence of miR-126 mimic and increased by miR-126 antagomir. These results suggest that miR-126 may modulate MLCK expression through the ERK/MAPK signaling pathway.

In conclusion, the present results suggest a role for miR-126 in palmitate-treated HUVECs cell migration by causing a downregulation in MLCK via ERK activation. Understanding how miR-126 regulate endothelial cell migration and cell apoptosis provide important insights into the development of AS.

## Data Availability Statement

All datasets presented in this study are included in the article/supplementary material.

## Author Contributions

YW and HZ study conception and design. YW, PY, and QZ acquisition of data. YW, MW, XZ, and LZ analysis and interpretation of the data. YW and HZ manuscript drafting and revision. All authors contributed to the article and approved the submitted version.

## Conflict of Interest

The authors declare that the research was conducted in the absence of any commercial or financial relationships that could be construed as a potential conflict of interest.
